# Warburg effect-promoted exosomal circ_0072083 releasing up-regulates NANGO expression through multiple pathways and enhances temozolomide resistance in glioma

**DOI:** 10.1186/s13046-021-01942-6

**Published:** 2021-05-11

**Authors:** Chenyu Ding, Xuehan Yi, Xiangrong Chen, Zanyi Wu, Honghai You, Xiaoyong Chen, Gaoqi Zhang, Yong Sun, Xingyao Bu, Xiyue Wu, Zhangya Lin, Jianjun Gu, Yuanxiang Lin, Dezhi Kang

**Affiliations:** 1 Department of Neurosurgery, The First Affiliated Hospital of Fujian Medical University, Fuzhou, 350001 Fujian People’s Republic of China; 2 Fujian Provincial Key Laboratory of Precision Medicine for Cancer, Fuzhou, 350001 Fujian People’s Republic of China; 3 Department of Otolaryngology Head and Neck Surgery, Fujian Medical University Union Hospital, Fuzhou, 350001 Fujian People’s Republic of China; 4 Department of Neurosurgery, The Second Affiliated Hospital, Fujian Medical University, Quanzhou, 362000 Fujian People’s Republic of China; 5 Department of Neurosurgery, Henan University People’s Hospital, Henan Provincial People’s Hospital, Zhengzhou, 450000 Henan People’s Republic of China; 6 Department of Neurosurgery, Zhengzhou University People’s Hospital, Henan Provincial People’s Hospital, Zhengzhou, 450000 Henan People’s Republic of China

**Keywords:** Glioma, Exosome, Temozolomide, Hsa_circ_0072083, miR-1252-5p, NANOG

## Abstract

**Background:**

Temozolomide (TMZ) resistance limits its application in glioma. Exosome can carry circular RNAs (circRNAs) to regulate drug resistance via sponging microRNAs (miRNAs). miRNAs can control mRNA expression by regulate the interaction with 3’UTR and methylation. Nanog homeobox (NANOG) is an important biomarker for TMZ resistance. Hitherto, it is unknown about the role of exosomal hsa_circ_0072083 (circ_0072083) in TMZ resistance in glioma, and whether it is associated with NANOG via regulating miRNA sponge and methylation.

**Methods:**

TMZ-resistant (*n* = 36) and sensitive (*n* = 33) patients were recruited. The sensitive cells and constructed resistant cells were cultured and exposed to TMZ. circ_0072083, miR-1252-5p, AlkB homolog H5 (ALKBH5) and NANOG levels were examined via quantitative reverse transcription polymerase chain reaction and western blot. The half maximal inhibitory concentration (IC50) of TMZ, cell proliferation, apoptosis, migration and invasion were analyzed via Cell Counting Kit-8, colony formation, flow cytometry, wound healing and transwell assays. The in vivo function was assessed using xenograft model. The N6-methyladenosine (m6A) level was analyzed via methylated RNA immunoprecipitation (MeRIP). Target relationship was investigated via dual-luciferase reporter assay and RNA immunoprecipitation. Warburg effect was investigated via lactate production, glucose uptake and key enzymes expression. Exosome was isolated and confirmed via transmission electron microscopy and specific protein expression.

**Results:**

circ_0072083 expression was increased in TMZ-resistant glioma tissues and cells. circ_0072083 knockdown restrained the resistance of resistant cells via decreasing IC50 of TMZ, proliferation, migration, invasion and xenograft tumor growth and increasing apoptosis. circ_0072083 silence reduced NANOG expression via blocking ALKBH5-mediated demethylation. circ_0072083 could regulate NANOG and ALKBH5 via targeting miR-1252-5p to control TMZ resistance. Warburg effect promoted the release of exosomal circ_0072083 in resistant cells. Exosomal circ_0072083 from resistant cells increased the resistance of sensitive cells to TMZ in vitro and xenograft model. Exosomal circ_0072083 level was enhanced in resistant patients, and it had a diagnostic value and indicated a lower overall survival in glioma.

**Conclusion:**

Exosomal circ_0072083 promoted TMZ resistance via increasing NANOG via regulating miR-1252-5p-mediated degradation and demethylation in glioma.

**Supplementary Information:**

The online version contains supplementary material available at 10.1186/s13046-021-01942-6.

## Highlights

1. hsa_circ_0072083 expression is increased and hsa_circ_0072083 knockdown decreases TMZ resistance in glioma.
2. hsa_circ_0072083 controls NANOG expression by regulating miR-1252-5p -mediated degradation and methylation via targeting ALKBH5.
3. The secretion of exosomal hsa_circ_0072083 is dependent on Warburg effect.
4. Exosomal hsa_circ_0072083 is associated resistance development

## Introduction

Glioma is a type of primary brain tumor with poor prognosis [1]. Chemotherapy is one of the main therapeutic strategies, and temozolomide (TMZ), an oral alkylating agent, is widely accepted as an option for chemotherapy of glioma of all grades [2]. TMZ treatment significantly increases the survival of patients, while the development of resistance limits its efficacy in clinic [3]. Hence, exploring the mechanism of TMZ resistance development in glioma is of importance.

The enrichment of extracellular vesicles with cargoes is to respond to TMZ treatment in glioma [4]. Exosome is a common type of extracellular vesicles with nanostructures, which can carry the cargos like noncoding RNAs to participate in the development and treatment of glioma [5, 6]. Circular RNAs (circRNAs) are a class of noncoding RNAs mainly formed via connection of the downstream and upstream exons, which have important roles in glioma [7]. Lots of circRNAs are dysregulated, and they can interact with microRNA (miRNA)/mRNA axes to be involved in tumorigenesis of glioma [8]. Moreover, exosomes can transfer circRNAs to control TMZ resistance in glioma, such as circRNA nuclear factor I X (circNFIX) and homeodomain interacting protein kinase 3 (circHIPK3), [9, 10].CircRNA hsa_circ_0072083 (circ_0072083) is derived from zinc finger RNA binding protein (ZFR), which may play a promoting or suppressive role in human tumors, such as gastric cancer and papillary thyroid cancer [11, 12]. However, whether circ_0072083 can be transferred via exosomes and its function on TMZ resistance in glioma are unknown.

miRNAs are a type of noncoding RNAs (~ 22 nucleotides) that can control mRNA degradation via pairing of 2–8 nucleotides of their sequence to the 3’untranslated region (UTR) of mRNA, which are related to the development and TMZ resistance in glioma [13, 14].miR-1252-5p is an anti-tumor miRNA in human tumor such as non-small cell lung cancer and papillary thyroid cancer [15, 16], and this miRNA is associated with drug resistance like paclitaxel in ovarian cancer [17]. Nevertheless, the role and mechanism of miR-1252-5p in TMZ resistance in glioma remain unclear. Nanog homeobox (NANOG) is a key stemness marker, which regulates tumor development [18]. NANOG contributes to tumor cell growth and TMZ resistance in glioma [19, 20]. N6-methyladenosine (m6A) is a common mRNA modification, and it is involved in the regulation of noncoding RNAs on tumorigenesis [21]. AlkB homolog H5 (ALKBH5) is a representative m6A demethylase which can lead to the demethylation of mRNA in human disease [22]. ALKBH5 maintains tumorigenicity of glioma stem-like cells [23]. Furthermore, a previous report suggests that ALKBH5 can control NANOG expression via regulating m6A demethylation [24]. Bioinformatics analysis displays miR-1252-5p might interact with circ_0072083,NANOG and ALKBH5 using Circinteractome (https://circinteractome.nia.nih.gov) and starBase (https://starbase.sysu.edu.cn). However, their interaction in glioma is not reported in the current study.

In this research, we aimed to study the function of exosomal circ_0072083 on TMZ resistance, and explore the regulatory network of circ_0072083/miR-1252-5p/ NANOG via competitive sponge and ALKBH5-mediated demethylation pathways in glioma. This study might provide new insight into the mechanism of TMZ resistance in glioma.

## Materials and methods

### Patients and samples

Authorized via the Ethics Committee of First Affiliated Hospital of Fujian Medical University and the Ethics Committee of Henan Provincial People’s Hospital, glioma patients received TMZ medication (75 mg/m2 once a day via intravenous injection) before surgery were recruited. The TMZ-resistant patients were those with progressive disease or recurrent disease within 6 months after the chemotherapy; while the TMZ-sensitive patients indicated those with recurrence > 6 months or no recurrence. TMZ-resistant patients (*n* = 36) and sensitive patients (*n* = 33) were recruited from First Affiliated Hospital of Fujian Medical University and Henan Provincial People’s Hospital, and they all provided the informed written consent. The clinical formation of patients was shown in Table 1. The tumor tissues from resistant or sensitive patients were determined via two experienced pathologists and frozen in liquid nitrogen. The serum samples were also collected from all patients by centrifugation at 1600 g for 10 min, and used for exosome isolation. A 36-months follow-up was performed for analysis of overall survival. This research was in line with the Helsinki Declaration.

**Table 1:** The correlation between circ_0072083 level and clinicopathological characteristics in glioma patients

Characteristics	All patients	Relative circ_0072083 level		*P* value
		High	Low	
Gender				> 0.05
Male	43	22	21	
Female	26	14	12	
Age (years)				> 0.05
≥ 50	39	20	19	
< 50	30	16	14	
Tumor size (cm)				< 0.05
≥ 4	29	24	5	
< 4	40	12	28	
IDH1				> 0.05
Mutation	33	20	13	
Wild type	36	16	20	
WHO grade				< 0.05
I-II	31	6	25	
III-IV	38	30	8	
MGMT methylated				< 0.05
Yes	32	6	26	
No	37	30	7	
TMZ-therapy				< 0.05
TMZ-sensitive	33	7	26	
TMZ-resistant	36	29	7	

### MGMT promotor methylation

O6-methylguanine-DNA methyltransferase (MGMT) is responsible for TMZ resistance, and MGMT promoter hypermethylation is a negative factor of TMZ resistance via promoting TMZ response by inducing MGMT silencing in glioma [25]. The level of MGMT promotor methylation was analyzed following the instruction of a previous report [26]. Based on the MTGT promotor sequence (GenBank number: X61657.1), the primer pairs (5′-TTTGTGTTTTGATGTTTGTAGGTTTTTGT-3′ and 5′-AACTCCACACTCTTCCAAAAACAAAACA-3′ for unmethylated MGMT; 93 bp) and (5′-TTTCGACGTTCGTAGGTTTTCGC-3′ and 5′-GCACTCTTCC GAAAACG AAACG-3′ for methylated MGMT; 81 bp) were designed and generated via Sangon (Shanghai, China). DNA from TMZ-resistant or sensitive tissues was isolated, and used for PCR response. The PCR was performed with the procedure: 95 °C for 12 min, and 40 cycles of 95 °C for 15 s, 59 °C for 30 s and 72 °C for 30 s, followed via 72 °C for 5 min. The PCR product was suffered from 2% agarose gel electrophoresis, and the methylated MGMT was determined via the visualized product using the methylated primer. The proportion of MGMT promoter methylation was expressed as methylated MGMT / (methylated MGMT + unmethylated MGMT) × 100%.

### Cell culture and establishment of TMZ-resistant cell lines

The glioma cell lines (U251 and U87), and 293 T cells were purchased from Procell (Wuhan, China), and maintained in Dulbecco’s Modified Eagle’s Medium (Thermo Fisher Scientific, Waltham, MA, USA) with 10% fetal bovine serum (Gibco, Gran Island, NY, USA) and 1% penicillin/streptomycin (Beyotime, Shanghai, china) at 5% CO_2_ and 37 °C. The medium was changed every 3 days.

The resistant cells were established according to a previous study with some modifications [27]. U251 and U87 cells (2 × 10^5^) were continuously exposed to increasing concentrations of TMZ (Selleck, Shanghai, China) until resistance to 50 μg/mL TMZ. The established TMZ-resistant cell lines were designated as U251/TR and U87/TR.

### RNA extraction and quantitative reverse transcription polymerase chain reaction (qRT-PCR)

RNA from tissues or cells was isolated using Trizol (Vazyme, Nanjing, China). The RNA in nucleus or cytoplasm was extracted with a Cytoplasmic & Nuclear RNA Purification kit (Norgen Biotek, Thorold, Canada). The exosomal RNA was prepared via using Exosome Purification and RNA Isolation kit (AmyJet Scientific, Wuhan, China). circRNA was purified via digesting the linear RNA using RNase R (Geneseed, Guangzhou, China). One μg RNA was used to complementary DNA synthetization using the miRNA Reverse Transcriptase kit or M-MLV Reverse Transcriptase kit (Thermo Fisher Scientific). The complementary DNA was mixed with SYBR (TaKaRa, Otsu, Japan) and specific primers (Sangon) for qRT-PCR. The procedure was set as: 95 °C for 5 min, and 40 cycles of 95 °C for 10 s, 58 °C for 30 s and 72 °C for 1 min. The primer sequences were displayed in Supplementary Table 1. U6 or 18 s rRNA functioned as a normalized reference. Relative RNA level was analyzed via 2^-ΔΔCt^ method.

### Circular structure of circRNA analysis

The circular of circ_0072083 was predicted via CircView (https://gb.whu.edu.cn/CircView/).

The circular structure of circRNA was analyzed via treatment of Actinomycin D and RNase R. 5 × 10^5^ U251/TR and U87/TR cells were challenged via 2 μg/mL Actinomycin D (Sigma, St. Louis, MO, USA) for 0, 6, 12 or 24 h. Next, RNA was isolated, and circ_0072083 and ZFR levels were detected via qRT-PCR.

For analysis of RNase R, the isolated RNA was incubated with 4 U/μg RNase R for 30 min. Then circ_0072083 and ZFR levels were detected via qRT-PCR.

### Cell transfection

Small interfering RNA (siRNA) for circ_0072083 (si-circ_ circ_0072083#1, si- circ_0072083#2 and si- circ_0072083#3), siRNA negative control (si-NC), short hairpin RNA (shRNA) for circ_0072083 (sh-circ_0072083), shRNA for ALKBH5 (sh-ALKBH5), shRNA negative control (sh-NC), miR-1252-5p mimic, mimic negative control (miR-NC), miR-1252-5p inhibitor (anti-miR-1252-5p), and inhibitor negative control (anti-NC) were generated via Ribobio (Guangzhou, China). The oligonucleotide sequences were exhibited in Supplementary Fig. 2. Cell transfection was performed into U251/TR and U87/TR cells via Lipofectamine 3000 (Thermo Fisher Scientific) for 24 h.

### Cell counting Kit-8 (CCK-8)

The half maximal inhibitory concentration (IC50) of TMZ and cell proliferation were investigated via CCK-8. For detection of IC50 of TMZ, 1 × 10^4^ cells were placed into 96-well plates, and exposed to different doses (0, 6.25, 12.5, 25, 50, 100, 200, 400 and 800 μg/mL) of TMZ for 24 h. Next, 10 μL CCK-8 (Solarbio, Beijing, China) was added into each well, and cells were cultured for 3 h. The optical density (OD) value was detected at 450 nm using a microplate reader (Bio-Rad, Hercules, CA, USA). Cell viability was normalized to the control group (0 μg/mL of TMZ), and the IC50 of TMZ was determined according to the viability curve.

For analysis of cell proliferation, 1 × 10^4^ cells were added into the 96-well plates, and incubated with 50 μg/mL of TMZ for 24, 48 or 72 h. At each time point, 10 μL CCK-8 was injected, and cells were incubated for 3 h. The OD value at 450 nm was determined through a microplate reader.

### Colony formation analysis

The colony ability was analyzed via colony formation analysis. Five hundred cells were inoculated into 6-well plates and incubated with 50 μg/mL of TMZ. After 10 days, the clones were fixed and stained with 0.5% crystal violet (Solarbio). Next, colony formation was imaged and the number was calculated.

### Flow cytometry

Cell apoptosis was detected with an Annexin V-fluorescein isothiocyanate (FITC) apoptosis detection kit (Sigma). 2 × 10^5^ cells were added into 12-well plates and cultured in medium containing 50 μg/mL of TMZ for 72 h. Subsequently, cells were harvested and interacted with Annexin V binding buffer, followed by dyeing to 10 μL Annexin V-FITC and propidium iodide (PI) for 5 min. Cells were examined with a flow cytometer (Beckman Coulter, Brea, CA, USA). The percentage of apoptotic cells (Annexin V-FITC^+^ and PI^+/−^) was calculated.

### Wound healing and transwell analyses

Cell migration and invasion were analyzed via wound healing and transwell analyses. For wound healing analysis, 2 × 10^5^ cells were added into 6-well plates, and grown to 90% confluence. Then a straight wound was induced using a 200-μL pipette tips, and cells were incubated with medium containing 50 μg/mL of TMZ for 24 h. The images were recorded at 0 and 24 h under a 100 × magnification microscope (Olympus, Tokyo, Japan). The wound healing ratio was expressed as (wound width at 0 h – wound width at 24 h)/wound width at 0 h × 100%.

For transwell migration analysis, 24-well transwell chambers (Corning Inc., Corning, NY, USA) with a fibronectin-coated polycarbonate membrane were used. 1 × 10^5^ cells were cultured in serum-free medium and added into the upper chambers. Five hundred μL medium containing 10% serum was added to the lower chambers. After treatment of 50 μg/mL of TMZ for 24 h, cells passed the membrane were fixed and dyed with 0.5% crystal violet. Cells were imaged under a 100 × magnification microscope, and cell number was calculated using Image J v1.8 (NIH, Bethesda, MD, USA). For transwell invasion analysis, the transwell chambers were precoated via Matrigel (Solarbio). 5 × 10^5^ cells were inoculated into the upper chambers, and the other procedures were same to migration analysis.

### Animal experiment

The animal experiments were approved via the Animal Ethics Committee of the Second Affiliated Hospital of Fujian Medical University / the Animal Ethical Committee of Henan Provincial People's Hospital. The BALB/c nude mice (male, 5-week-old) were purchased from Vital River (Beijing, China), and maintained in a specific condition. U251/TR cells (2 × 10^6^ per mouse) with stable transfection of sh-circ_0072083 or sh-NC were subcutaneously injected into mice. After 7 days, mice were intraperitoneally injected with 20 mg/kg TMZ or equal volume of phosphate buffer saline (PBS; Solarbio) solution twice a week. The mice were divided into four groups (sh-NC + PBS, sh-circ_0072083 + PBS, sh-NC + TMZ, and sh-circ_0072083 + TMZ; *n* = 5 per group). Tumor volume was measured every 7 days with a formula (0.5 × length × width^2^). After 28 days, mice were euthanized, and tumor tissues were weighed and collected for RNA isolation.

Moreover, the exosomes isolated from U251/TR cells stably transfected with sh-NC or sh-circ were termed as U251/TR-sh-NC EXO or U251/TR-sh-circ_0072083 EXO. U251 cells (2 × 10^6^ per mouse) were subcutaneously injected into mice. After 7 days, the mice were intraperitoneally injected with 20 mg/kg TMZ and intratumorally injected with 10 μg U251/TR-sh-NC EXO or U251/TR-sh-circ_0072083 EXO or PBS. The mice were divided into three groups (PBS + TMZ, U251/TR-sh-NC EXO + TMZ, and U251/TR-sh-circ_0072083 EXO + TMZ; *n* = 5 per group). Tumor volume was detected, and mice were euthanized after 28 days. Tumor weight was measured, and the tumor tissues were used for RNA or protein isolation.

### Western blot

Protein was extracted using radioimmunoprecipitation assay buffer (Thermo Fisher Scientific), and concentration was determined with a bicinchoninic acid kit (Beyotime). Twenty μg protein was separated via sodium dodecyl sulfate-polyacrylamide gel electrophoresis, and transferred on polyvinylidene fluoride membrane (Bio-Rad). After being blocked in 3% bovine serum albumin (Solarbio), the membranes were interacted with primary antibodies for ALKBH5 (ab244296, 1:2000 dilution, Abcam, Cambridge, UK), NANOG (ab203919,1:1000 dilution, Abcam), cluster of differentiation (CD)63 (ab216130, 1:1000 dilution, Abcam), CD81 (ab109201, 1:5000 dilution, Abcam),tumor susceptibility gene 101 (TSG101) (ab125011, 1:2000 dilution, Abcam), Golgi marker 130 (GM130) (ab187514, 1:2000 dilution, Abcam), glucose transporter 1 (GLUT1) (ab128033, 1:3000 dilution, Abcam), lactate dehydrogenase A (LDHA) (ab84716, 1:1000 dilution, Abcam), pyruvate kinase M2 (PKM2) (ab137852, 1:1000 dilution, Abcam) or β-actin (ab179467, 1:5000 dilution, Abcam) overnight and horseradish peroxidase-labeled secondary antibody IgG (ab97080, 1:8000 dilution, Abcam) for 2 h. β-actin functioned as a normalized control. After incubation of enhanced chemiluminescence reagent (Beyotime), the visualized blots were analyzed via Image J v1.8. Relative protein level was expression as fold-change of the control group.

### Exosome isolation, validation, exposure and stability

Cell medium or serum samples were collected and used for exosome isolation using a total exosome RNA and protein isolation kit (Thermo Fisher Scientific) following the instructions [28]. The exosomes were resuspended in PBS and validated via transmission electron microscopy (TEM) and levels of exosomal markers (CD63, CD81 and TSG101). For TEM analysis, the exosomes were fixed with 5% glutaraldehyde (Sigma) and then observed under a TEM (Hitachi, Tokyo, Japan) after the sample preparation as previously reported [29]. The size distribution and concentration of exosomes were analyzed via ZetaView (Particle Metrix, Ammersee, Germany). The exosomes isolated from U251/TR and U87/TR cells stably transfected with sh-NC or sh-circ_0072083 were termed as U251/TR-sh-NC EXO, U251/TR-sh-circ_0072083 EXO, U87/TR-sh-NC EXO or U87/TR-sh-circ_0072083 EXO. For co-incubation with the sensitive cells, U251 and U87 cells were incubated with 2 μg/mL of exosomes for 24 h. For stability testing, the exosomes from serum were exposed to different conditions, including the incubation for 0, 6, 12 and 24 h, and varying pH values (HCl or NaOH treatment), followed via detection of exosomal circ_0072083 level.

### Warburg effect analysis

The Warburg effect was investigated via lactate production, glucose uptake and levels of glycolysis key enzymes. 2 × 10^6^ cells were collected and lysed, and the lysates were used for detection of lactate production and glucose uptake using the specific lactate assay kit or glucose assay kit (Abcam) according to the instructions of manufacturer. Relative levels of lactate production and glucose uptake were normalized to the control group. The abundances of glycolysis key enzymes (GLUT1, LDHA and PKM2) were detected via qRT-PCR and western blot. To stimulate or block the Warburg effect, U251/TR and U87/TR cells were incubated with 5 ng/mL of TNF-α (stimulus) or 1 μM Shikonin (inhibitor) for 24 h [30]. Cells challenged via PBS were used as controls.

### Bioinformatics analysis, dual-luciferase reporter assay, RNA immunoprecipitation (RIP) and methylated RIP (MeRIP)

The targets of circ_0072083 were predicted via Circinteractome (https://circinteractome.nia.nih.gov), and the miRNAs that could target NANOG and ALKBH5 were predicted via starBase (https://starbase.sysu.edu.cn). The sequence (… UCCUUC …) of circ_0072083 or 3′ UTR of NANOG and ALKBH5 with miR-1252-5p complementary site was cloned into the pGL3 vector (Promega, Madison, WI, USA), generating the wild-type (WT) luciferase reporter vector WT-circ_0072083, WT-NANOG 3’UTR and WT-ALKBH5 3’UTR.The mutant (MUT) luciferase reporter vectors MUT-circ_0072083, MUT-NANOG 3’UTR and MUT-ALKBH5 3’UTR were constructed via mutating the complementary site of miR-1252-5p. The luciferase reporter vectors and miR-1252-5p mimic or miR-NC were co-transfected into 293 T cells (a tool cell line for dual-luciferase reporter assay) for 24 h. The luciferase activity was examined with a luciferase analysis kit (Promega).

RIP analysis was conducted using a Magna RIP kit (Sigma). 1 × 10^7^ U251/TR and U87/TR cells were lysed, and incubated with 50 μL beads pre-coated with antibodies for argonaute 2 (anti-Ago2) (ab186733, 1:50 dilution, Abcam), anti-ALKBH5 (ab244296, 1:30 dilution, Abcam) or anti-IgG (ab190475, 1:100 dilution, Abcam) for overnight. The enrichment abundances of circ_0072083, miR-1252-5p, NANOG and ALKBH5 on the beads were detected via qRT-PCR. The methylated RNA immunoprecipitation (MeRIP) was performed using N6-methyladenosine (m6A) antibody (ab208577, 1:30 dilution, Abcam), and other procedures were same to RIP analysis. The enrichment level of NANOG was measured by qRT-PCR.

### Statistical analysis

GraphPad Prism 8 (GraphPad Inc., La Jolla, CA, USA) was used for statistical analysis. The experiments were performed with 3 biological replicates × 3 technical replicates, unless otherwise indicated. Data were expressed as mean ± standard deviation (SD). Overall survival of patients was assessed via Kaplan-Meier plot and log-rank test. The association between exosomal circ_0072083 level and clinicopathologic features of patients was tested via χ^2^ test. The difference was compared by Student *t*-test (for 2 groups) or analysis of variance (ANOVA) followed via Tukey’s or Sidak’s post hoc test (for multiple groups). It was statistically significant when *P* < 0.05.

## Results

### circ_0072083 abundance is enhanced in TMZ-resistant glioma tissues and cells

**Fig. 1:**
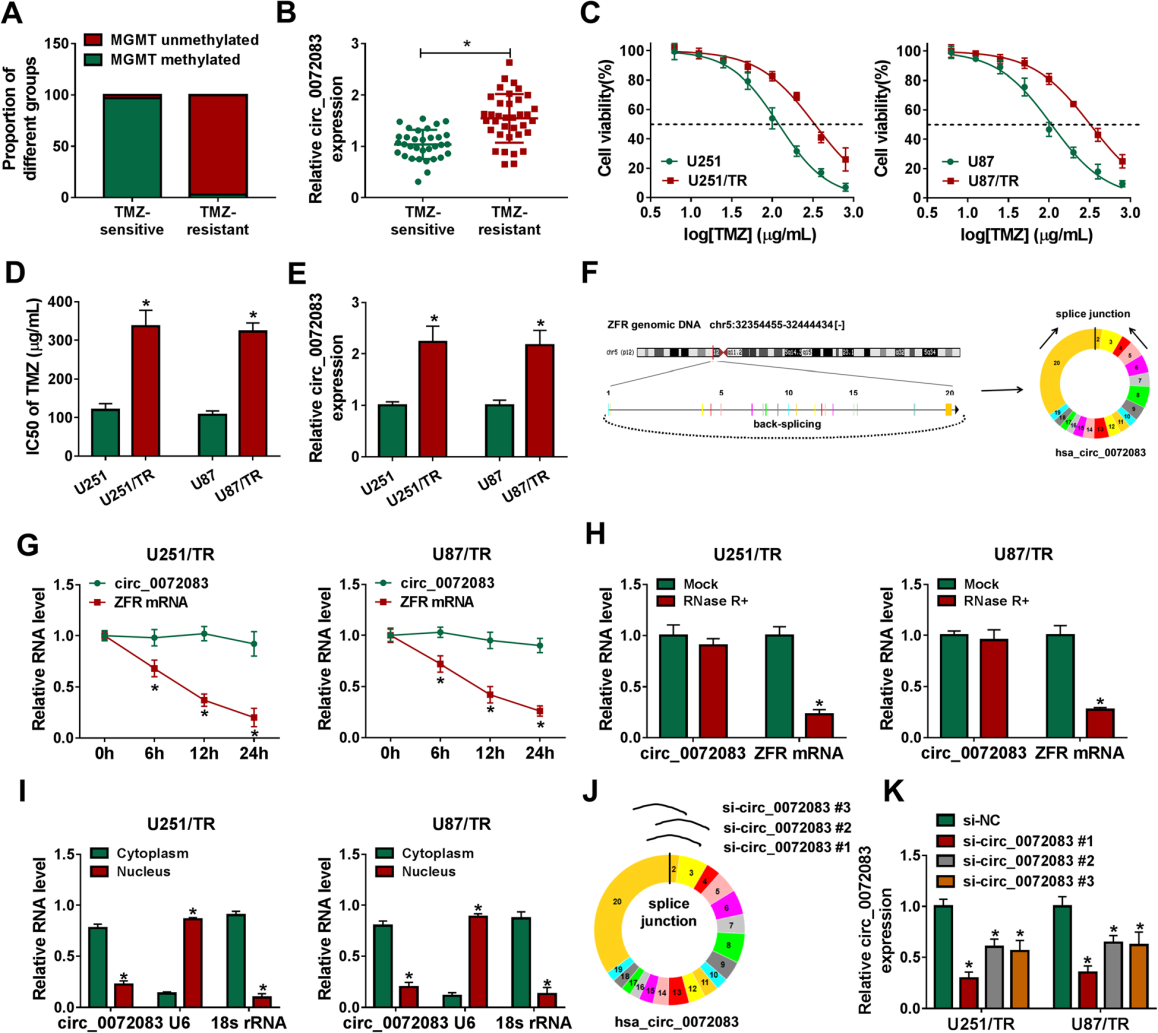
circ_0072083 expression is increased in TMZ-resistant glioma tissues and cells. **a** The proportion of MGMT methylation was analyzed in TMZ-resistant or sensitive tissues. **b** circ_0072083 level was detected via qRT-PCR in TMZ-resistant (*n* = 36) or sensitive tissues (*n* = 33). **c** and **d** Cell viability and IC50 of TMZ were measured via CCK-8 in cells after treatment of various doses of TMZ. **e** circ_0072083 abundance was examined in TMZ-resistant or sensitive cells. **f** The information of circ_0072083 was analyzed via CircView. **g** and **h** circ_0072083 and ZFR mRNA levels were detected via qRT-PCR after treatment of Actinomycin D for different time points or RNase R. **i** circ_0072083 abundance was examined via qRT-PCR in cytoplasm and nucleus with U6 and 18 s rRNA as references. **j** and **k** The si-circ_0072083#1, si-circ_0072083#2 and si-circ_0072083#3 were constructed by targeting the splice junction, and their knockdown efficacy was detected via qRT-PCR. ^*^P < 0.05

The TMZ-resistant tissues were confirmed with lower proportion of MGMT promoter methylation than TMZ-sensitive samples (Fig. 1a). To explore whether circ_0072083 was involved in TMZ resistance in glioma, we measured the expression change of circ_0072083 in the resistant and sensitive tissues. As displayed in Fig. 1b, circ_0072083 abundance was higher in TMZ-resistant tissues (*n* = 36) than that in sensitive tissues (*n* = 33) (Fig. 1b). Moreover, we constructed the TMZ-resistant cell lines (U251/TR and U87/TR), which had higher IC50 of TMZ than the sensitive cells (Fig. 1c and d). circ_0072083 abundance was increased more than 2-fold in the resistant cells (U251/TR and U87/TR) than sensitive cells (U251 and U87) (Fig. 1e). Additionally, the information and structure of circ_0072083 were analyzed. CircView software showed that circ_0072083 was generated via back-splicing of exon 2 to exon 20 of ZFR transcripts (Fig. 1f). Furthermore, circ_0072083 was more resistant to Actinomycin D and RNase R than corresponding linear type (ZFR) in the two cell lines, indicating the circular structure of circ_0072083 (Fig. 1g and h). In addition, circ_0072083 was mainly expressed in cytoplasm in U251/TR and U87/TR cells (Fig. 1i). To knock down circ_0072083 in cells, three siRNAs (si-circ_0072083#1, si-circ_0072083#2 and si-circ_0072083#3) targeted the splice junction of circ_0072083 were constructed, and their efficacy was confirmed (Fig. 1j and k).

### circ_0072083 knockdown reduces TMZ resistance in TMZ-resistant glioma cells

**Fig. 2:**
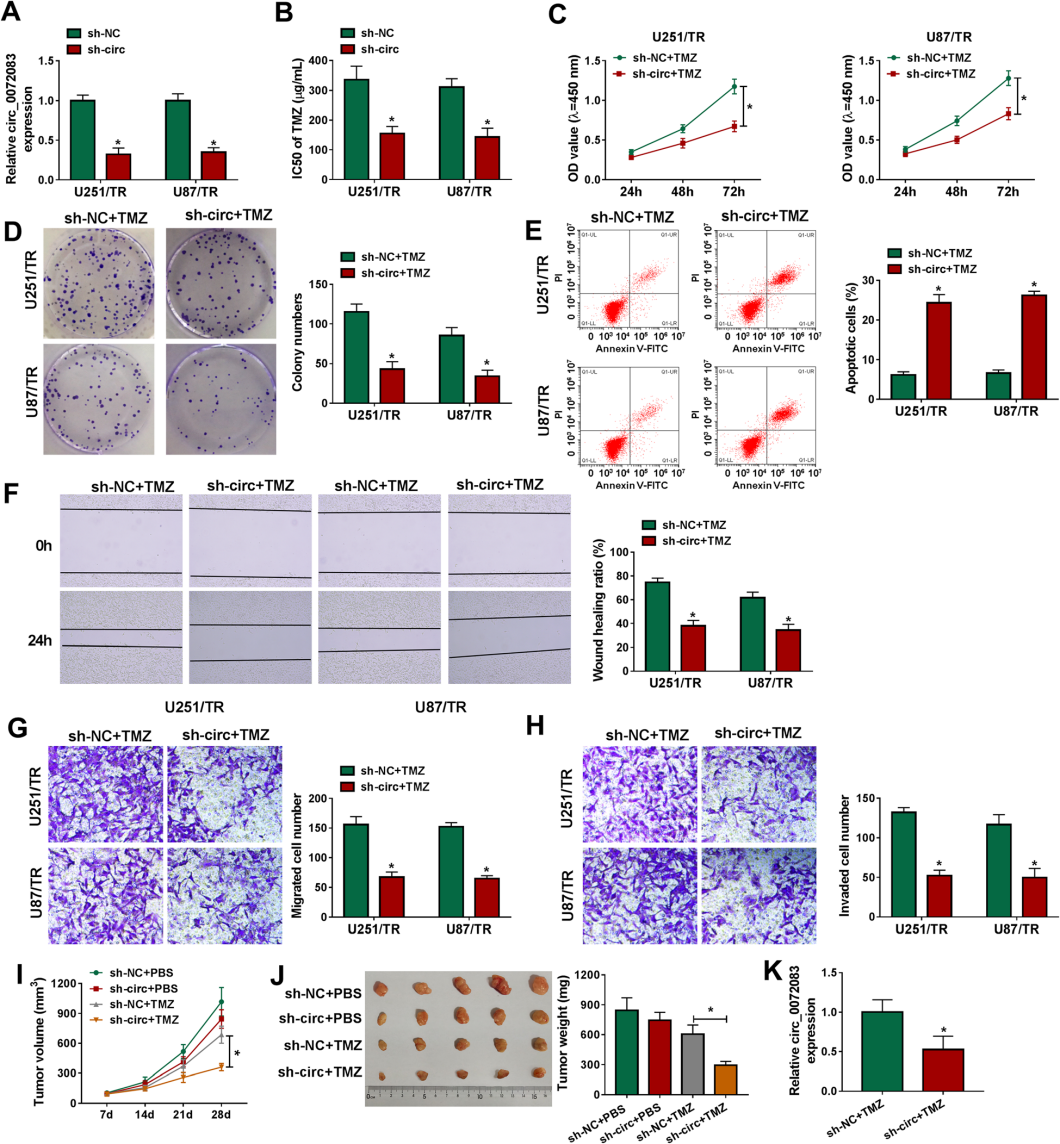
circ_0072083 silence decreases TMZ resistance in the resistant glioma cells. **a** circ_0072083 expression was detected via qRT-PCR in resistant cells transfected with sh-circ_0072083 or sh-NC. **b** IC50 of TMZ was analyzed via CCK-8 in cells with transfection of sh-circ_0072083 or sh-NC. Cell proliferation **c**, colony ability **d**, apoptosis **e**, migration **f** and **g** and invasion **h** were measured by CCK-8, colony formation, flow cytometry, wound healing and transwell analyses respectively in resistant cells transfected with sh-circ_0072083 or sh-NC after treatment of 50 μg/mL of TMZ. **i** and **j** Tumor volume and weight were detected in each group (*n* = 5). Xenograft model was established using U251/TR cells transfected with sh-circ_0072083 or sh-NC, and then treated via PBS or TMZ. **k** circ_0072083 abundance was measured via qRT-PCR in tumor tissues of each group. ^*^P < 0.05

To study the function of circ_0072083 on TMZ resistance, U251/TR and U87/TR cells were transfected with sh-circ_0072083 or sh-NC. The sh-circ_0072083 for stable transfection was constructed on the basis of the sequence of si-circ_0072083#1 with highest knockdown efficacy. Transfection of sh-circ induced more than 65% reduction of circ_0072083 level in U251/TR and U87/TR cells (Fig. 2a). circ_0072083 knockdown evidently decreased the IC50 of TMZ in U251/TR and U87/TR cells (Fig. 2b). Next, functional analyses were performed in cells with treatment of TMZ (50 μg/mL). In the presence of TMZ, circ_0072083 silence significantly repressed cell proliferation via decreasing the abilities of proliferation and colony formation (Fig. 2c and d). Moreover, circ_0072083 interference clearly increased the apoptosis of U251/TR and U87/TR cells in the presence of TMZ (Fig. 2e). In addition, circ_0072083 knockdown markedly impaired the abilities of migration and invasion in the two cell lines challenged via TMZ (Fig. 2f-h). Furthermore, the effect of circ_0072083 on TMZ efficacy was investigated in a xenograft model. U251/TR cells transfected with sh-circ_0072083 or sh-NC were used to establish the xenograft model, and then mice were treated via TMZ (20 mg/kg) or PBS. Tumor volume and weight were clearly decreased in sh-circ + TMZ group compared with sh-NC + TMZ group (Fig. 2i and j). Additionally, circ_0072083 abundance was significantly reduced in sh-circ_0072083 + TMZ group in comparison to sh-NC + TMZ group (Fig. 2k). These results showed that circ_0072083 silence attenuates TMZ resistance in glioma.

### circ_0072083 silence inhibits NANOG level via regulating ALKBH5-mediated demethylation in TMZ-resistant glioma cells

**Fig. 3:**
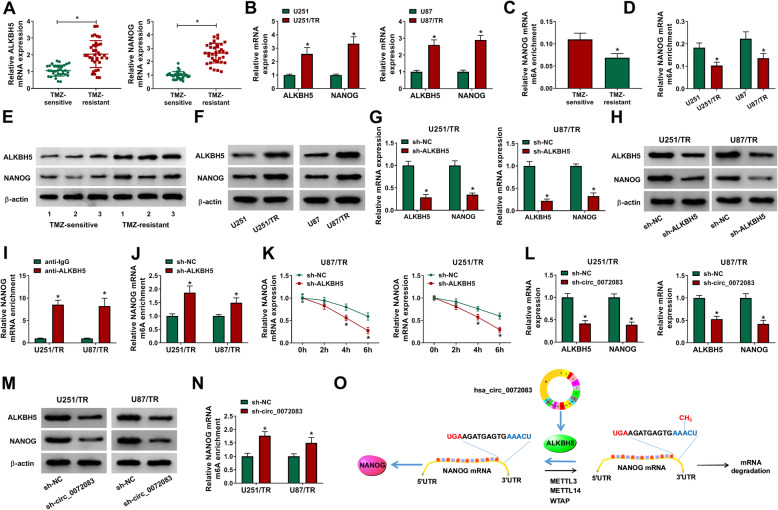
circ_0072083 knockdown decreases NANOG expression by regulating ALKBH5-mediated demethylation. **a** ALKBH5 and NANOG mRNA levels were detected via qRT-PCR in TMZ-resistant (*n* = 36) or sensitive tissues (*n* = 33). **b** ALKBH5 and NANOG levels were measured by qRT-PCR in TMZ-resistant and sensitive cells. **c** and **d** NANOG mRNA m6A enrichment level was analyzed in TMZ-resistant or sensitive tissues and cells. **e** and **f** ALKBH5 and NANOG protein levels were examined by western blot in TMZ-resistant or sensitive tissues and cells. **g** and **h** ALKBH5 and NANOG levels were examined via qRT-PCR and western blot in resistant cells transfected with sh-ALKBH5 or sh-NC. **i** NANOG mRNA enrichment level was detected in resistant cells after RIP assay using anti-ALKBH5 or anti-IgG. **j** NANOG mRNA m6A enrichment level was examined in resistant cells transfected with sh-ALKBH5 or sh-NC. **k** NANOG mRNA level was measured via qRT-PCR in cells with transfection of sh-ALKBH5 or sh-NC after treatment of Actinomycin D for different time points. **l** and **m** ALKBH5 and NANOG levels were detected via qRT-PCR and western blot in cells transfected with sh-circ_0072083 or sh-NC. **n** NANOG mRNA m6A enrichment level was detected in cells transfected with sh-circ_0072083 or sh-NC. **o** The schematic diagram of circ_0072083-regulated NANOG through ALKBH5-mediated demethylation. ^*^P < 0.05

To explore whether ALKBH5 and NANOG were required via circ_0072083 in regulating TMZ resistance, their levels were detected in TMZ-resistant tissues and cells. As shown in Fig. 3a and b, ALKBH5 and NANOG mRNA levels were significantly elevated in TMZ-resistant tissues and cells compared with those in the sensitive group. Moreover, NANOG mRNA m6A level was evidently decreased in TMZ-resistant group in comparison to that in sensitive group (Fig. 3c and d). In addition, ALKBH5 and NANOG protein levels were evidently increased in TMZ-resistant tissues and cells (Fig. 3e and f). And NANOG expression was markedly reduced via ALKBH5 silence using transfection of sh-ALKBH5 (Fig. 3g and h). Furthermore, RIP assay displayed that NANOG could be enriched by ALKBH5 in U251/TR and U87/TR cells (Fig. 3i). Additionally, ALKBH5 silence evidently enhanced m6A level of NANOG mRNA and decreased NANOG mRNA stability in response to Actinomycin D (Fig. 3j and k). Besides, the effect of circ_0072083 on ALKBH5 and NANOG expression was evaluated in U251/TR and U87/TR cells. Results exhibited that circ_0072083 silence significantly decreased ALKBH5 and NANOG abundances and increased the m6A level of NANOG mRNA (Fig. 3l-n). The schematic diagram was exhibited in Fig. 3o, which showed a multiprotein complex (METTL3, METTL14 and WTAP) mediated m6A modification of NANOG mRNA to induce its degradation. circ_0072083 could upregulate ALKBH5 expression, which mediated demethylation of NANOG mRNA 3’UTR, leading to the increased NANOG mRNA stability. These data indicated that circ_0072083 could regulate NANOG expression via regulating ALKBH5-mediated demethylation.

### circ_0072083 interacts with miR-1252-5p to modulate ALKBH5/NANOG axis and TMZ resistance in TMZ-resistant glioma cells

**Fig. 4:**
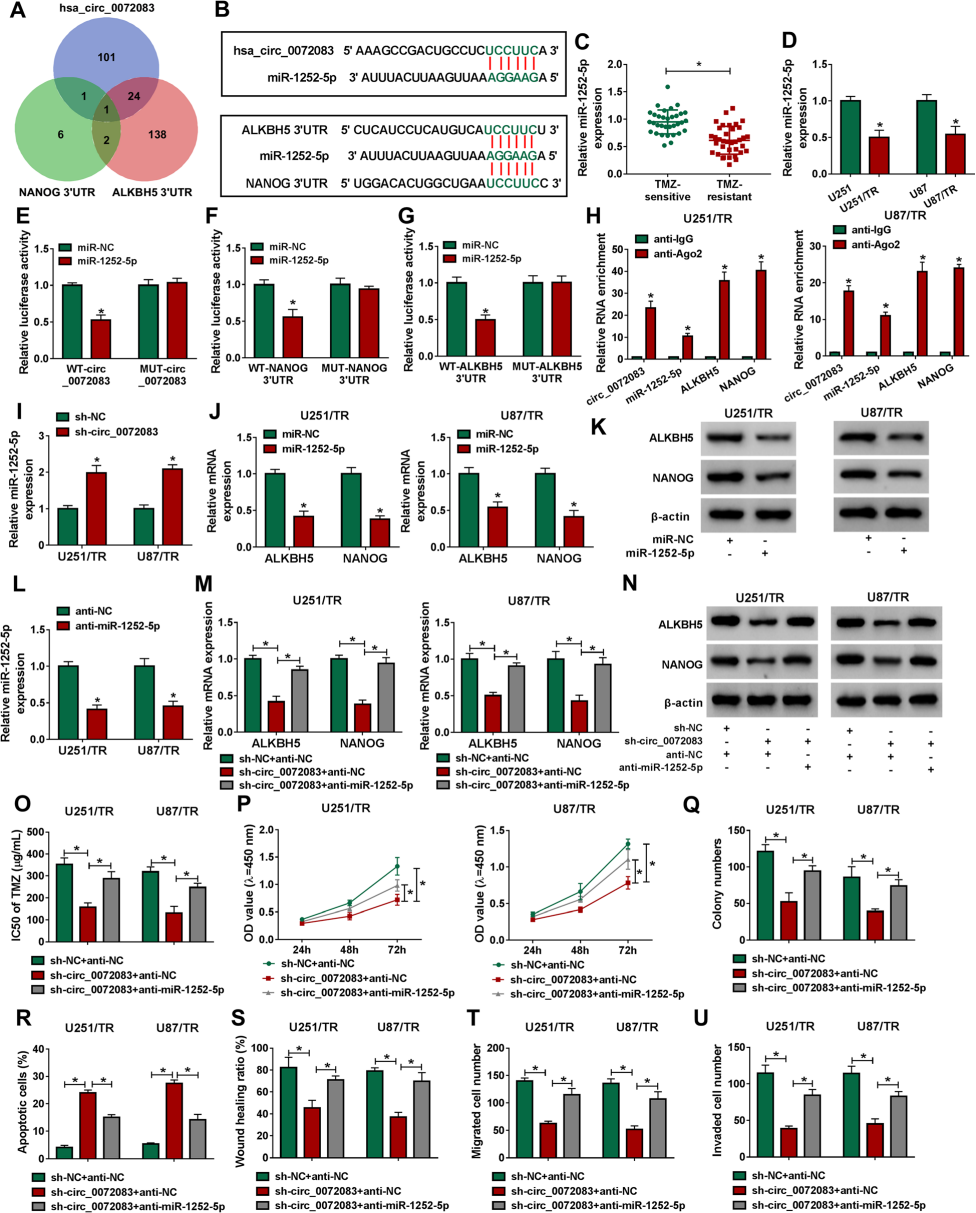
circ_0072083 targets miR-1252-5p to regulate ALKBH5/NANOG and TMZ resistance in the resistant glioma cells. **a** The potential miRNA that interacted with circ_0072083, ALKBH5 and NANOG was analyzed via Circinteractome and starBase. **b** The binding site of miR-1252-5p and circ_0072083, ALKBH5 and NANOG was exhibited. **c** miR-1252-5p expression were detected via qRT-PCR in TMZ-resistant (*n* = 36) or sensitive tissues (*n* = 33). **d** miR-1252-5p abundance was measured by qRT-PCR in TME-resistant and sensitive cells. **e**-**g** Luciferase activity was detected in 293 T cells with transfection of miR-1252-5p mimic or miR-NC and the constructed luciferase reporter vectors. **h** miR-1252-5p, circ_0072083, ALKBH5 and NANOG levels were detected after RIP assay using anti-Ago2 or anti-IgG. **i** miR-1252-5p expression was measured via qRT-PCR in cells transfected with sh-circ or sh-NC. (J and K) ALKBH5 and NANOG levels were detected via qRT-PCR and western blot in cells transfected with miR-1252-5p mimic or miR-NC. **l** miR-1252-5p expression was measured via qRT-PCR in cells transfected with anti-miR-1252-5p or anti-NC. **m** and **n** ALKBH5 and NANOG levels were examined via qRT-PCR and western blot in cells transfected with sh-NC + anti-NC, sh-circ_0072083 + anti-NC or sh-circ_0072083 + anti-miR-1252-5p. IC50 of TMZ **o**, cell proliferation **p**, colony ability **q**, apoptosis **r**, migration **s** and **t** and invasion **u** were measured by CCK-8, colony formation, flow cytometry, wound healing and transwell analyses respectively in resistant cells transfected with sh-NC + anti-NC, sh-circ_0072083 + anti-NC or sh-circ_0072083 + anti-miR-1252-5p after treatment of the indicated concentration of TMZ. ^*^P < 0.05

To analyze how circ_0072083 regulate ALKBH5/NANOG axis, the potential miRNA as a crosstalk was explored. Based on the database of Circinteractome and starBase, the Venn diagram showed that only one miRNA (miR-1252-5p) could bind with circ_0072083, ALKBH5 and NANOG (Fig. 4a), and the complementary sequences (… UCCUUC …) were displayed in Fig. 4b. miR-1252-5p expression was remarkably decreased in TMZ-resistant tissues and cells (Fig. 4c and d). To validate their interaction, the wild-type and mutant luciferase reporter vectors were constructed. miR-1252-5p overexpression obviously decreased the luciferase activity in the wild-type group (WT-circ_0072083, WT-NANOG 3’UTR and WT-ALKBH5 3’UTR), while it did not change the activity in the mutant group (MUT-circ_0072083, MUT-NANOG 3’UTR and MUT-ALKBH5 3’UTR) (Fig. 4e-g). Moreover, Ago2 RIP assay showed that miR-1252-5p, circ_0072083, ALKBH5 and NANOG could be enriched on the same complex (Fig. 4h). In addition, miR-1252-5p level was evidently enhanced via circ_0072083 silence in U251/TR and U87/TR cells (Fig. 4i). Furthermore, the effect of miR-1252-5p on ALKBH5 and NANOG levels was analyzed in cells transfected with miR-1252-5p mimic or miR-NC. The efficacy of miR-1252-5p mimic was validated in Supplementary Fig. 1. miR-1252-5p overexpression significantly decreased ALKBH5 and NANOG levels in U251/TR and U87/TR cells (Fig. 4j and k). Additionally, the effect of circ_0072083/miR-1252-5p axis on ALKBH5 and NANOG levels was investigated in cells transfected with sh-NC + anti-NC, sh-circ_0072083 + anti-NC or sh-circ_0072083 + anti-miR-1252-5p. The efficacy of anti-miR-1252-5p was confirmed in Fig. 4l. ALKBH5 and NANOG levels was notably reduced via circ_0072083 silence, which were restored by miR-1252-5p knockdown (Fig. 4m and n). Besides, miR-1252-5p knockdown reversed silence of circ_0072083-mediated reduction of IC50 of TMZ, cell proliferation, migration and invasion and promotion of apoptosis in U251/TR and U87/TR cells (Fig. 4o-u). These results suggested that circ_0072083 could regulate miR-1252-5p/ALKBH5 /NANOG axis to control TMZ resistance in glioma cells.

### The secretion of exosomal circ_0072083 is dependent on Warburg effect in TMZ-resistant glioma cells

**Fig. 5:**
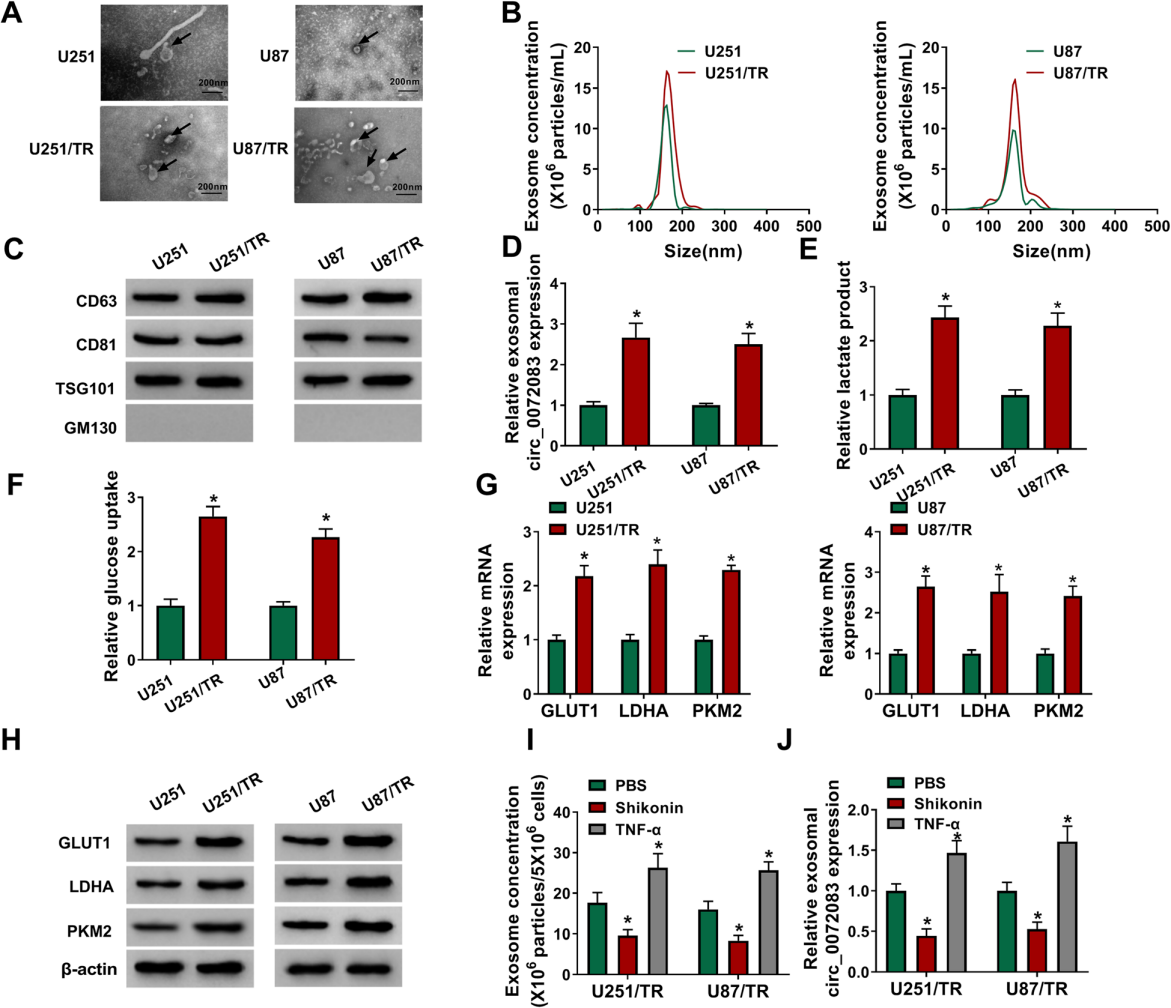
The release of exosome is associated with Warburg effect in resistant glioma cells. **a** The secretion of exosome was confirmed via TEM. Scale bar: 200 nm. **b** The concentration and size of exosome were analyzed. **c** CD63, CD81, TSG101 and GM130 protein levels were detected via western blot in exosome from cells. **d** Exosomal circ_0072083 level was detected via qRT-PCR in resistant or sensitive cells. **e** and **f** Lactate production and glucose uptake levels were measured in resistant or sensitive cells. **g** and **h** GLUT1, LDHA and PKM2 levels were examined via qRT-PCR and western blot in resistant or sensitive cells. **i** and **j** Exosome concentration and exosomal circ_0072083 level were examined in resistant cells after stimulation of Shikonin, TNF-α or PBS. ^*^P < 0.05

To explore whether circ_0072083 was carried via exosomes, the exosomes were isolated from medium of TMZ-resistant and sensitive cells. The exosomes were confirmed via TEM in Fig. 5a. The size was mainly at 100–200 nm, and resistant cells had higher concentration of exosome than sensitive cells (Fig. 5b). The exosomes were also identified via the presence of specific markers (CD63, CD81 and TSG101) and absence of Golgiosome marker (GM130) (Fig. 5c). Additionally, exosomal circ_0072083 level was evidently elevated in U251/TR and U87/TR cells compared with that in U251 and U87 cells (Fig. 5d). Furthermore, higher Warburg effect was found in the resistant cells than sensitive cells, which was revealed via the increased levels of lactate production, glucose uptake and key enzymes (GLUT1, LDHA and PKM2) (Fig. 5e-h). In addition, inhibition of Warburg effect (Shikonin) significantly decreased exosome concentration and exosomal circ_0072083 level, but promotion of Warburg effect (TNF-α) played an opposite role (Fig. 5i and j). Besides, exosome concentration was positive associated with glucose concentration and Warburg effect level (Supplementary 2A-E). Previous reports suggested the epidermal growth factor (EGF) and oleanolic acid (OA) could enhance or inhibit exosome secretion [30]. Here we found EGF and OA promoted or suppressed the Warburg effect in U251/TR and U87/TR cells (Supplementary Fig. 2F and G). Moreover, EGF-mediated promotion of exosome secretion was mitigated by inhibition of Warburg effect (Shikonin); and OA-mediated inhibition of exosome secretion was reversed via promotion of Warburg effect (TNF-α) (Supplementary Fig. 2H and I). These data indicated the release of exosomal circ_0072083 in TMZ-resistant glioma cells was dependent on the Warburg effect.

### Exosomal circ_0072083 from resistant cells promotes TMZ resistance in sensitive glioma cells

**Fig. 6:**
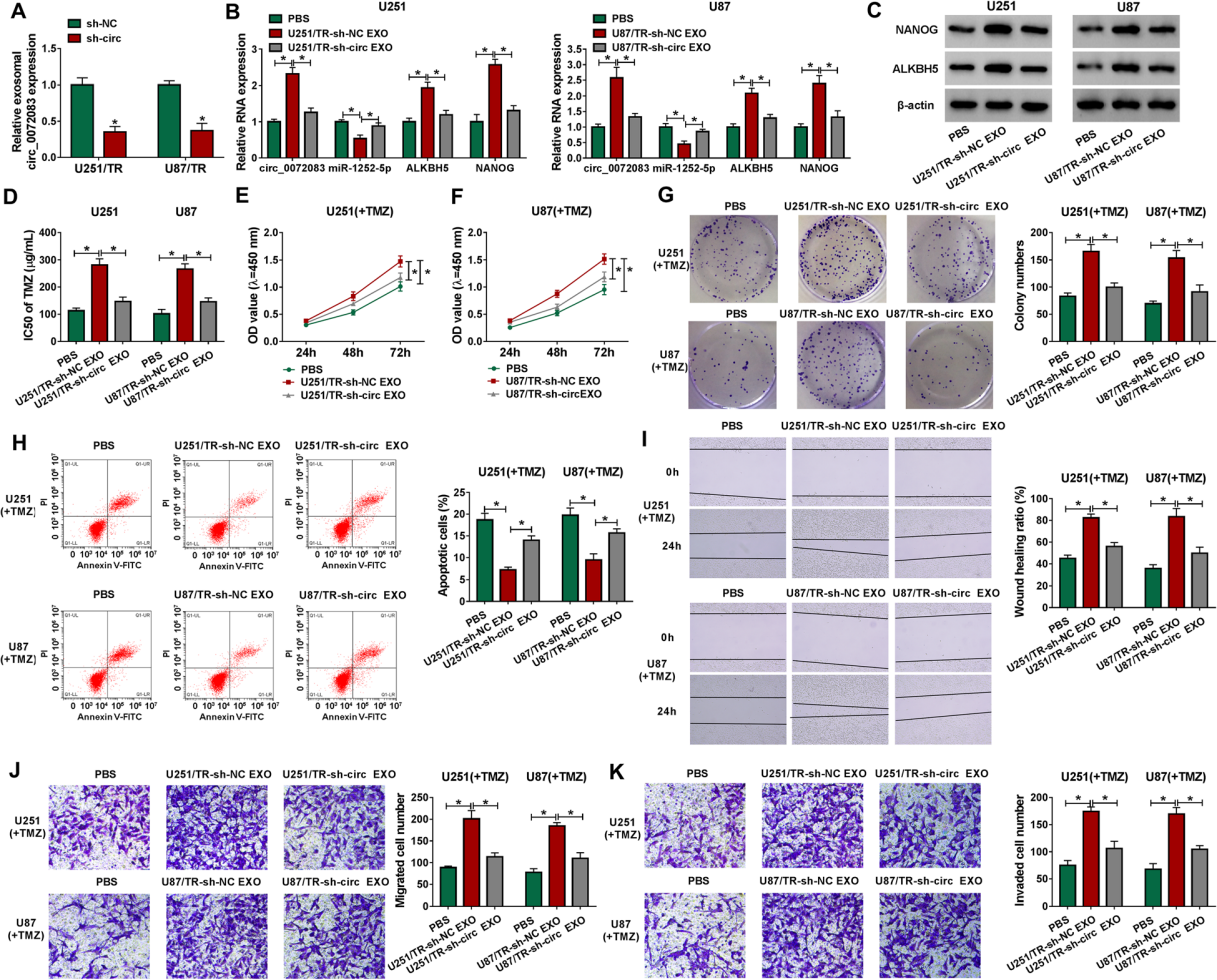
Exosomal circ_0072083 from resistant cells increases the resistance of sensitive glioma cells. **a** Exosomal circ_0072083 level was detected by qRT-PCR in resistance cells transfected with sh-circ_0072083 or sh-NC. **b** and **c** circ_0072083, miR-1252-5p, ALKBH5 and NANOG levels were examined in sensitive cells after treatment of exosome from resistant cells or PBS. IC50 of TMZ **d**, cell proliferation **e** and **f**, colony ability **g**, apoptosis **h**, migration **i** and **j** and invasion **k** were measured by CCK-8, colony formation, flow cytometry, wound healing and transwell analyses respectively in sensitive cells with stimulation of exosome from resistant cells or PBS after treatment of the indicated concentration of TMZ. ^*^P < 0.05

**Fig. 7:**
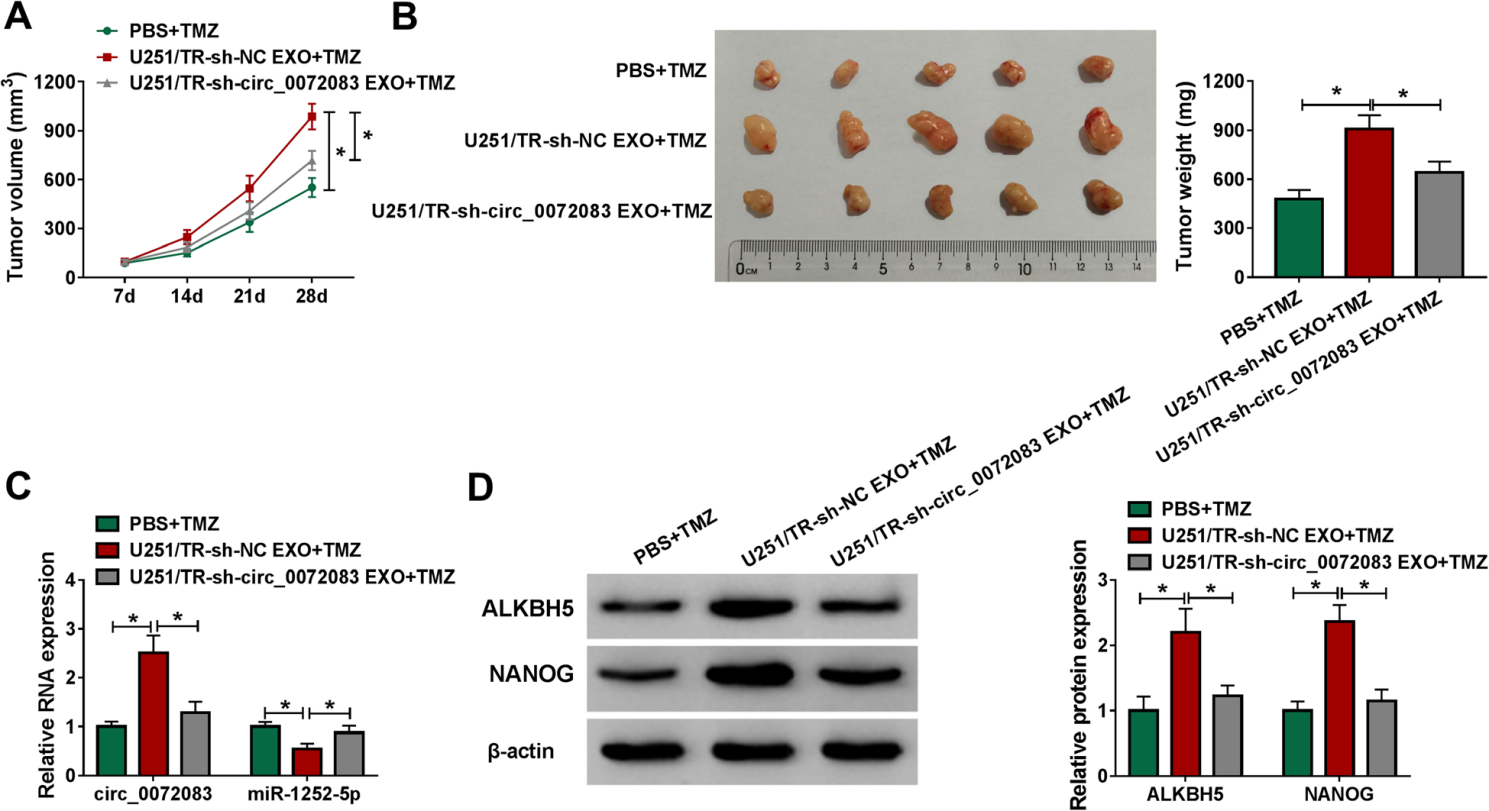
Exosomal circ_0072083 from resistant cells increases sensitive glioma cell growth in a xenograft model under TMZ. U251 cells were used to establish the xenograft model, and then mice were treated via exosome from U251/TR cells with transfection of sh-circ_0072083 or sh-NC and TMZ (*n* = 5). **a** and **b** Tumor volume and wight were detected in each group. **c** and **d** circ_0072083, miR-1252-5p, ALKBH5 and NANOG levels were examined in tumor tissues of each group. ^*^P < 0.05

To study the function of exosomal circ_0072083 on TMZ resistance, the exosomes were isolated from U251/TR and U87/TR cells transfected with sh-circ_0072083 (U251/TR-sh-circ_0072083 EXO and U87/TR-sh-circ_0072083 EXO) or sh-NC (U251/TR-sh-NC EXO and U87/TR-sh-NC EXO), and incubated with U251 and U87 cells. Exosomal circ_0072083 level was evidently decreased in the medium of U251/TR and U87/TR cells with transfection of sh-circ (Fig. 6a). The levels of circ_0072083, ALKBH5 and NANOG were significantly increased and miR-1252-5p expression was reduced in U251 and U87 cells with incubation of U251/TR-sh-NC EXO and U87/TR-sh-NC EXO compared with PBS group, while these events were attenuated via circ_0072083 down-regulation in cells with treatment of U251/TR-sh-circ_0072083 EXO and U87/TR-sh-circ_0072083 EXO (Fig. 6b and c). Furthermore, incubation of U251/TR-sh-NC EXO and U87/TR-sh-NC EXO significantly enhanced the IC50 of TMZ in U251 and U87 cells, which was weakened via circ_0072083 knockdown (Fig. 6d). Additionally, introduction of U251/TR-sh-NC EXO and U87/TR-sh-NC EXO evidently promoted cell proliferation, migration and invasion and inhibited apoptosis of U251 and U87 cells under treatment of TMZ (50 μg/mL), but these events were relieved by circ_0072083 silence in U251/TR-sh-circ_0072083 EXO and U87/TR-sh-circ_0072083 EXO groups (Fig. 6e-k). Moreover, the effect of exosomal circ_0072083 on glioma cell growth in vivo was investigated via U251 cell xenograft model. The xenograft mice were treated via PBS + TMZ (20 mg/kg), U251/TR-sh-NC EXO (10 μg) + TMZ (20 mg/kg), or U251/TR-sh-circ_0072083 EXO (10 μg) + TMZ (20 mg/kg). Tumor volume and weight were clearly increased via addition of U251/TR-sh-NC EXO, which were mitigated by introduction of U251/TR-sh-circ_0072083 EXO (Fig. 7a and b). In addition, the abundances of circ_0072083, ALKBH5 and NANOG were evidently enhanced and miR-1252-5p expression was decreased in tumor tissues in U251/TR-sh-NC EXO + TMZ group, and these events were reversed in U251/TR-sh-circ_0072083 EXO + TMZ group (Fig. 7c and d). These results indicated that exosomal circ_0072083 increased TMZ resistance in sensitive cells.

### Exosomal circ_0072083 is related to TMZ resistance, diagnosis and survival of glioma patients

**Fig. 8:**
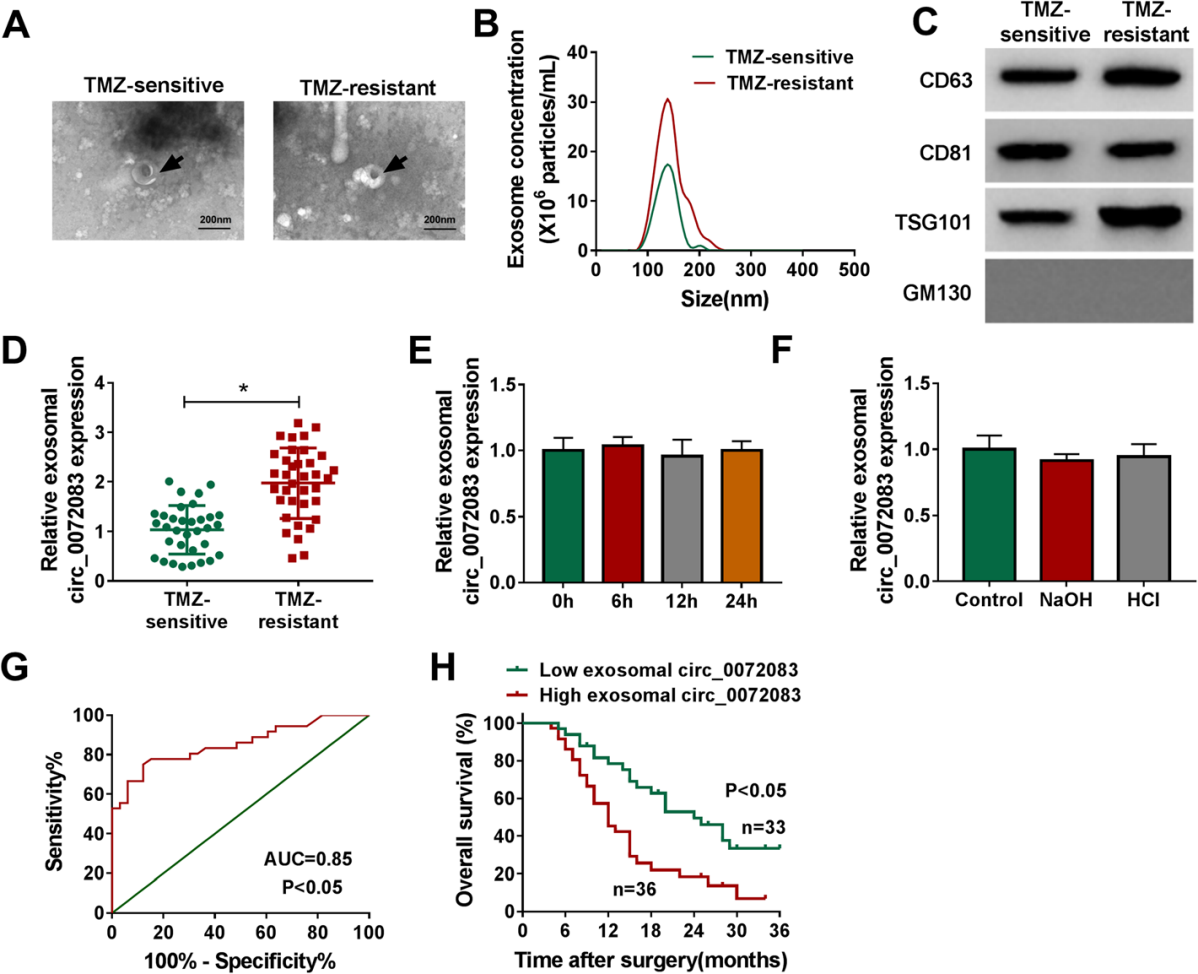
Exosomal circ_0072083 is associated with TMZ resistance, diagnosis and survival of glioma patients. **a** The secretion of exosome was confirmed via TEM in serum of TMZ-resistant or sensitive patients. Scale bar: 200 nm. **b** The concentration and size of exosome from patients were analyzed. **c** CD63, CD81, TSG101 and GM130 protein levels were examined via western blot in exosome from patients. **d** Exosomal circ_0072083 expression was detected via qRT-PCR in resistant or sensitive patients. **e** and **f** Exosomal circ_0072083 level was measured after incubation of different time points or treatment of different pH values. **g** The diagnostic value of exosomal circ_0072083 was analyzed using a ROC curve. **h** The overall survival of patients was analyzed in low or high exosomal circ_0072083 group. ^*^P < 0.05

**Fig. 9:**
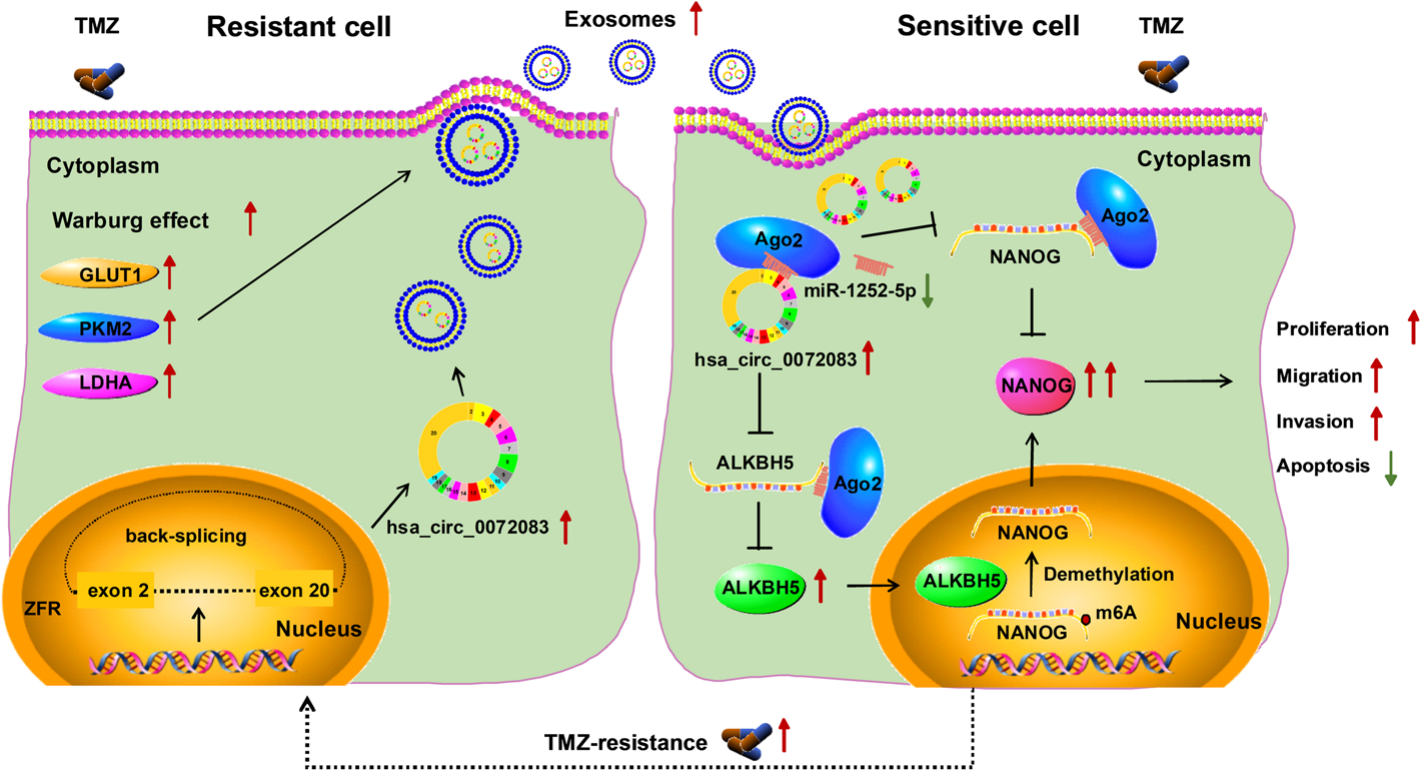
The schematic diagram of this study. Under Warburg effect, exosome from TMZ-resistant cells could transfer circ_0072083 to communicate with sensitive cells, further to target miR-1252-5p/NANOG axis by regulating ALKBH5-mediated demethylation, thus leading to resistance development of sensitive cells

To analyze the value of exosomal circ_0072083 in glioma patients, the exosomes were isolated from patients’ serum samples. The exosomes were validated via TEM and specific markers (CD63, CD81 and TSG101) (Fig. 8a and c). Moreover, the size was mainly at 100–200 nm, and its concentration was higher in resistant patients (Fig. 8b). Additionally, exosomal circ_0072083 level was significantly up-regulated in TMZ-resistant patients (*n* = 36) compared with TMZ-sensitive patients (*n* = 33) (Fig. 8d). Furthermore, the stability of exosomal circ_0072083 was evaluated via exposing these exosomes to different conditions, and the results showed the abundance of exosomal circ_0072083 in TMZ-resistant patients was not significantly affected via different incubation time and varying pH values (Fig. 8e and f). In addition, exosomal circ_0072083 could act as an independent diagnostic target for glioma (area under curve (AUC) = 0.85, *P* < 0.05), and patients with high exosomal circ_0072083 level had lower overall survival (*P* < 0.05) (Fig. 8g and h). The patients were divided into high (*n* = 36) or low (*n* = 33) exosomal circ_0072083 group according to the mean level. Table 1 summarized that high exosomal circ_0072083 level was associated with tumor size, advanced WHO grade, MGMT methylation and TMZ resistance in glioma patients. These findings suggested that exosomal circ_0072083 had an important role in glioma patients. The main mechanism of this study was displayed in Fig. 9, which showed that exosomal circ_0072083 could increase TMZ resistance in glioma via regulating miR-1252-5p/NANOG axis by modulating ALKBH5-mediated demethylation under Warburg effect (Fig. 9).

## Discussion

Glioma is a type of deadly brain tumor with huge challenge for cure [31]. TMZ chemotherapy is a main strategy for glioma treatment, and the resistance development is a limited factor for its application [32]. Therefore, we wanted to explore new strategies for ameliorating TMZ sensitivity in glioma. The exosomal noncoding RNAs (including exosomal circRNAs) play important roles in the development, treatment and regulation of drug resistance in glioma [6]. In this study, we mainly investigated the promoting effect of exosomal circ_0072083 on TMZ resistance, and validated the regulatory network of circ_0072083/miR-1252-5p/NANOG in glioma.

Liu et al. suggested that circ_0072083 could repress cell proliferation and promote apoptosis by regulating miR-130a/miR-107/phosphatase and tensin homolog (PTEN) in gastric cancer [11]. Moreover, Wei et al. reported that circ_0072083 could contribute to cell proliferation and invasion via regulating miR-1261/C8orf4 axis in papillary thyroid carcinoma [12]. In addition, circ_0072083 could promote cell proliferation, migration and invasion via regulating miR-101-3p/cullin 4B (CUL4B) [33], and facilitated cisplatin resistance via regulating miR-545-3p/Cbl proto-oncogene like 1 (CBLL1) in non-small cell lung cancer [34]. These reports indicated that circ_0072083 is a multifunctional circRNA in different tumors due to the varying tumor microenvironment. In this study, we found that high expression of circ_0072083 might be associated with TMZ resistance in glioma patients and cell lines. Furthermore, via in vitro and in vivo experiments, we found that circ_0072083 silence inhibited TMZ resistance in glioma, indicating this circRNA might function as a promising target for regulating TMZ treatment in glioma.

NANOG is a stemness regulatory factor, which modulates carcinogenesis and multidrug resistance in malignant tumors [35, 36]. Previous studies suggested that NANOG contributed to the glioma malignancy via promoting cell growth [20, 37]. Additionally, NANOG might be activated via the aberrant Notch signaling to promote tumor recurrence and invasion in glioma [38]. More importantly, multiple evidences have confirmed that NANOG could facilitate TMZ resistance in glioma [19, 39, 40]. Here we found circ_0072083 knockdown could decrease NANOG expression. Thus, we next wanted to explore how circ_0072083 could regulate NANOG. miRNAs could act as a crosstalk for the interaction of circRNA and mRNA [8], and they were associated with TMZ resistance in glioma [3]. miR-1252-5p has been reported as an anti-tumor miRNA [15–17], and is associated with regulation of drug resistance [17]. Our study was the first time to validate miR-1252-5p could interact with circ_0072083 and NANOG, thus resulting in that circ_0072083 could modulate NANOG indirectly via miR-1252-5p to regulate TMZ resistance in glioma.

m6A is also one important mRNA modification in glioma [41]. The m6A demethylase ALKBH5 is one of important factors for TMZ resistance in glioma [42]. Liu et al. reported that ALKBH5 could enhance sex determining region Y-box 2 (SOX2) expression via regulating the demethylation to increase TMZ resistance in glioma [43]. This suggested that ALKBH5 might regulate resistance-related mRNA to control TMZ resistance. Previous studies reported that ALKBH5 could up-regulate NANOG expression via regulating the demethylation of NANOG in different tumors, like breast cancer, oral squamous cell carcinoma and ovarian cancer [24, 44, 45]. Similarly, we also found that ALKBH5 could increase NANOG expression in glioma. Moreover, the m6A modification may be addressed by noncoding RNA [21]. Apart from the direct interaction of miR-1252-5p and 3’UTR of NANOG, we also found that miR-1252-5p could bind with 3’UTR of ALKBH5. Hence, we thought miR-1252-5p could directly target NANOG 3’UTR or inhibit ALKBH5-mediated demethylation to reduce NANOG expression in glioma.

CircRNAs can be carried via exosomes to exhibit their functions to promote or inhibit tumor development [46]. Moreover, exosome release was affected by TMZ treatment in glioma [4]. By isolating the exosomes from cell medium or patients’ serum, we found that circ_0072083 could be carried via exosomes. Multiple evidences showed that the release of exosomes was dependent on the Warburg effect [30, 47, 48]. Similarly, we also identified that the Warburg effect contributed to the transfer of exosomal circ_0072083 in glioma. Exosomes are responsible for the communication in microenvironment in glioma [49]. Here we confirmed that exosomal circ_0072083 from resistant cells might increase the resistance of TMZ to sensitive cell lines. In addition, we found the diagnostic and prognostic values of exosomal circ_0072083 in glioma patients. Collectively, the exosomal circ_0072083 might increase TMZ resistance and act as important target in glioma.

## Conclusions

In conclusion, exosomal circ_0072083 could promote TMZ resistance in glioma by increasing NANOG, possibly via regulating miR-1252-5p-mediated degradation and miR-1252-5p/ALKBH5 axis-mediated demethylation. This research provides a novel insight into the understanding of TMZ resistance, and indicates a new target for improving TMZ treatment in glioma.

## Supplementary Information

**Additional file 1: Table S1.** The primer sequences for qRT-PCR in this study.

**Additional file 2: Table S2.** The oligo sequences for transfection in this study.

**Additional file 3: Figure S1.**

**Additional file 4: Figure S2.**

## Data Availability

All data in our study are available upon request.

## Abbreviations

TMZ: Temozolomide
NANOG: Nanog homeobox
ALKBH5: AlkB homolog H5
MeRIP: Methylated RNA immunoprecipitation
ZFR: Zinc finger RNA binding protein
UTR: Untranslated region
MGMT: Methyltransferase
IC50: Inhibitory concentration
SOX2: Sex determining region Y-box 2
