# Supplementary material for: Warburg effect-promoted exosomal circ_0072083 releasing up-regulates NANGO expression through multiple pathways and enhances temozolomide resistance in glioma
Source: J Exp Clin Cancer Res. 2021 May 11;40:164. doi: 10.1186/s13046-021-01942-6 (PMC8111743; doi:10.1186/s13046-021-01942-6)
Supplement: Supplementary file 1 — Additional file 1: Table S1. The primer sequences for qRT-PCR in this study. [file 13046_2021_1942_MOESM1_ESM.docx]

**Supplementary Table 1. The primer sequences for qRT-PCR in this study.**

| Name | Sequence (5’-3’) | |
| --- | --- | --- |
|  | Forward | Reverse |
| miR-1252-5p | GCCGAGagaaggaaaTTgaaT | AGTGCAGGGTCCGAGGTATT |
| U6 | GCTCGCTTCGGCAGCACA | GAGGTATTCGCACCAGAGGA |
| circ_0072083 | AAACTACTTTTGCTTTGAAATGAT | GCTGTGGGTGAATCCAAAGTA |
| ZFR | GAGGAGTACTGGCGAAGACG | GTCAGGACGACGTAAGGGTG |
| ALKBH5 | AGTTCAGTCTTCTGCTCGCC | AGGAACTGTGGACATGGCAG |
| NANOG | TCCCCTAATTTGTTGGTTGTGC | TTCACAGATGCCTGCGTAGC |
| GLUT1 | GAACTCTTCAGCCAGGGTCC | TCACACTTGGGAATCAGCCC |
| LDHA | CAGGTGGTTGAGAGGGTCTT | CTTCAAACGGGCCTCTTCCT |
| PKM2 | CCCGATCAGTGGAGCTGAAG | AGGAAGTCGGCACCTTTCTG |
| 18S rRNA | ACCCGTTGAACCCCATTCGTGA | GCCTCACTAAACCATCCAATC |
