# Supplementary material for: Warburg effect-promoted exosomal circ_0072083 releasing up-regulates NANGO expression through multiple pathways and enhances temozolomide resistance in glioma
Source: J Exp Clin Cancer Res. 2021 May 11;40:164. doi: 10.1186/s13046-021-01942-6 (PMC8111743; doi:10.1186/s13046-021-01942-6)
Supplement: Supplementary file 2 — Additional file 2: Table S2. The oligo sequences for transfection in this study. [file 13046_2021_1942_MOESM2_ESM.docx]

**Supplementary Table 2. The oligo sequences for transfection in this study.**

| Name | Sequence (5’-3’) |
| --- | --- |
| si-circ_0072083#1 | CGGCUGGGCAUAAAUUUGAAU |
| si-circ_0072083#2 | AGCCGGCUGGGCAUAAAUUUG |
| si-circ_0072083#3 | UGGGCAUAAAUUUGAAUAUGA |
| si-NC | AAGACAUUGUGUGUCCGCCTT |
| sh-circ | GATCCCGGCTGGGCATAAATTTGAATCTCGAGATTC  AAATTTATGCCCAGCCGTTTTTG |
| sh-ALKBH5 | GATCC ATTCTTCAGCCCTAACAGGCACTCGAGTGCC  TGTTAGGGCTGAAGAAT TTTTTG |
| sh-NC | CCGGGCGCGATAGCGCTAATAATTTCTC |
| miR-1252-5p mimic | agaaggaaauugaauucauuua |
| miR-NC | CGAUCGCAUCAGCAUCGAUUGC |
| anti-miR-1252-5p | UAAAUGAAUUCAAUUUCCUUCU |
| anti-NC | CUAACGCAUGCACAGUCGUACG |
