# Supplementary figures and images for: Warburg effect-promoted exosomal circ_0072083 releasing up-regulates NANGO expression through multiple pathways and enhances temozolomide resistance in glioma
Source: J Exp Clin Cancer Res. 2021 May 11;40:164. doi: 10.1186/s13046-021-01942-6 (PMC8111743; doi:10.1186/s13046-021-01942-6)

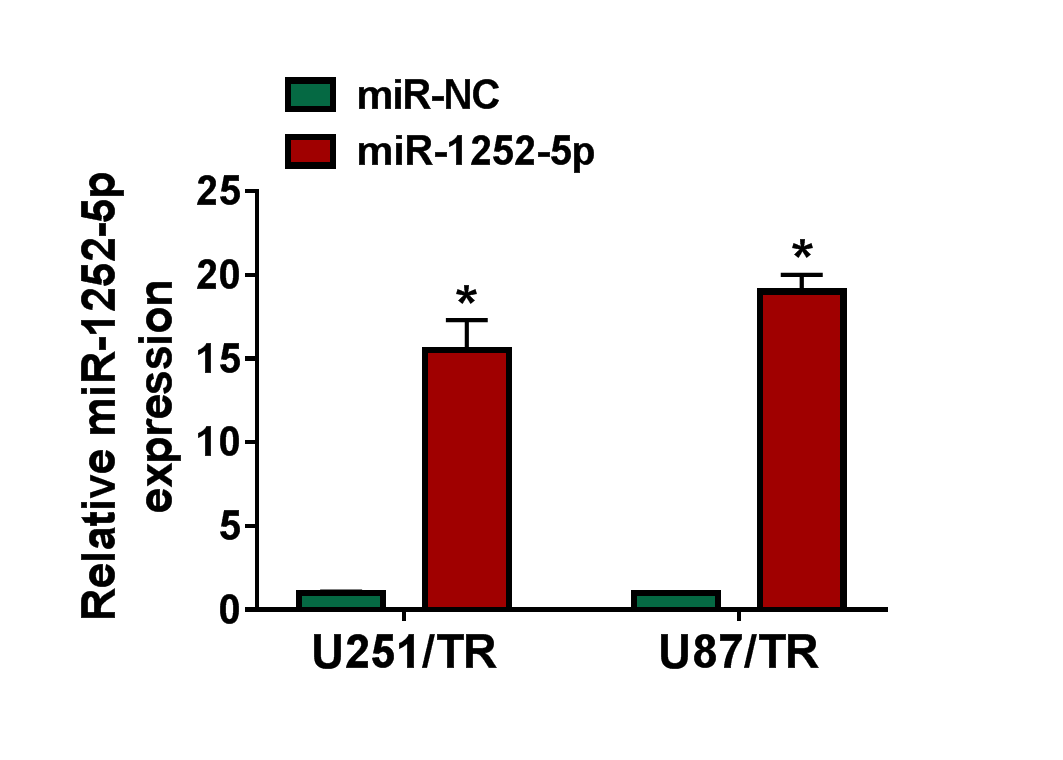

Supplement: Supplementary file 3 — Additional file 3: Figure S1. [file 13046_2021_1942_MOESM3_ESM.tif]

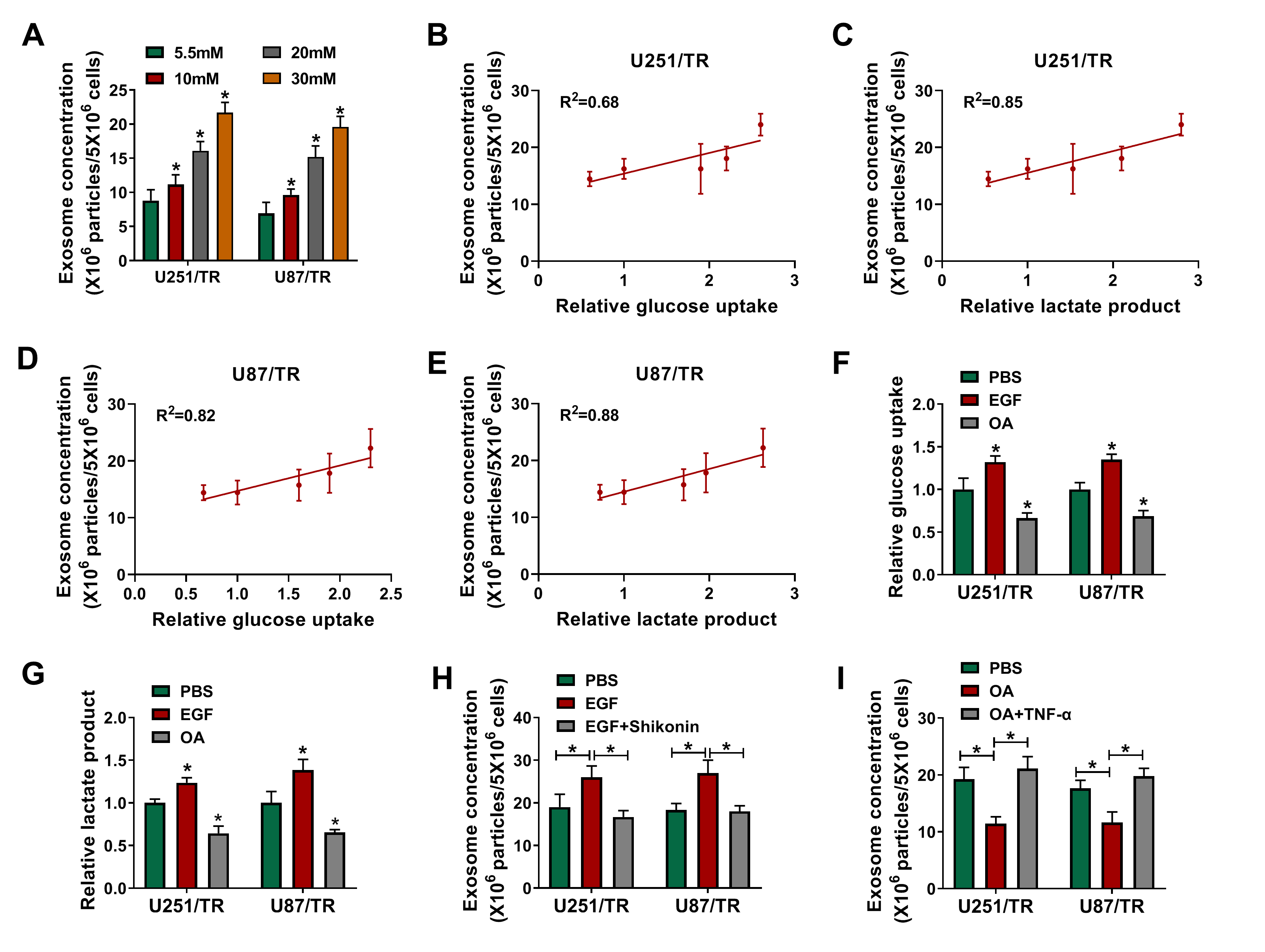

Supplement: Supplementary file 4 — Additional file 4: Figure S2. [file 13046_2021_1942_MOESM4_ESM.tif]
